# Outcome Analysis of Posterior Cruciate Ligament Injuries: A Narrative Review

**DOI:** 10.7759/cureus.47410

**Published:** 2023-10-20

**Authors:** Anmol Suneja, Sanjay V Deshpande, Hitendra Wamborikar, Swapnil V Date, Sachin Goel, Gursimran Sekhon

**Affiliations:** 1 Orthopaedics, Jawaharlal Nehru Medical College, Datta Meghe Institute of Higher Education and Research, Wardha, IND

**Keywords:** ligament, reconstruction, knee, injury, posterior cruciate ligament

## Abstract

The primary posterior stabilizer of the knee is the posterior cruciate ligament (PCL), the largest intra-articular ligament in the human knee. One of the four primary ligaments of the knee joint, the PCL, serves to support the tibia on the femur. An extreme force applied anteriorly to the proximal tibia of the flexed knee results in trauma to the PCL. Dashboard injuries, which occur when the knee is driven into the dashboard after a collision with a motor vehicle, are frequent causes. Grade 1 and 2 acute injuries are often addressed conservatively due to the PCL's natural capacity for mending. If a grade 3 injury occurs, a cautious trial can be conducted on elderly or low-demand patients. When standard treatment for isolated grade 3 injuries has failed, surgery is advised. Single-bundle or double-bundle techniques using either transtibial tunnel or tibial inlay techniques are among the reconstruction approaches. Restoring the natural kinematics of the knee and forestalling persistent posterior and mixed rotatory knee laxity are the ultimate goals of treating PCL injuries through a personalized strategy. These injuries may become more common in the future as more people participate in sports. As a result of ongoing instability, discomfort, diminished function, and the emergence of inflammatory and degenerative disorders of joints, PCL rips are becoming more well-acknowledged as a cause of morbidity and decreased function.

## Introduction and background

The posterior cruciate ligament (PCL) is among the strongest ligaments in the knee and serves as the main stabilizer for the posterior portion of the joint. In order to stop the posterior translation of the tibia and rotational instability, it closely collaborates with the components of the posterolateral corner (PLC) [1]. Injuries to other ligaments, menisci, or cartilage also occur in the majority of PCL injuries [2].

The incidence of PCL injuries in acute knee injuries has been reported to be as high as 44% [3]. It has been demonstrated that PCL insufficiency affects the knee's kinematics, causing instability and degenerative chondral alterations, particularly during functional activity. Posterior cruciate ligament injuries are classified as 'mild' when it is a partial tear of the PCL with minimal ligament damage; 'moderate' with more significant damage to ligament fibers; and 'severe' with a complete rupture of the PCL with a fully disrupted ligament.

Nonoperative care has been cited by authors as an effective option for the treatment of (mild or moderate) isolated grade 1 or 2 PCL rips or in individuals with less demanding physical conditions, and surgical intervention for patients with (severe) grade 3 PCL rips or when conservative treatment has proven ineffective. Nevertheless, the clinical results of surgical care are still inconsistent, and many patients still have knee instability and pain [4-8].

**Figure 1:**
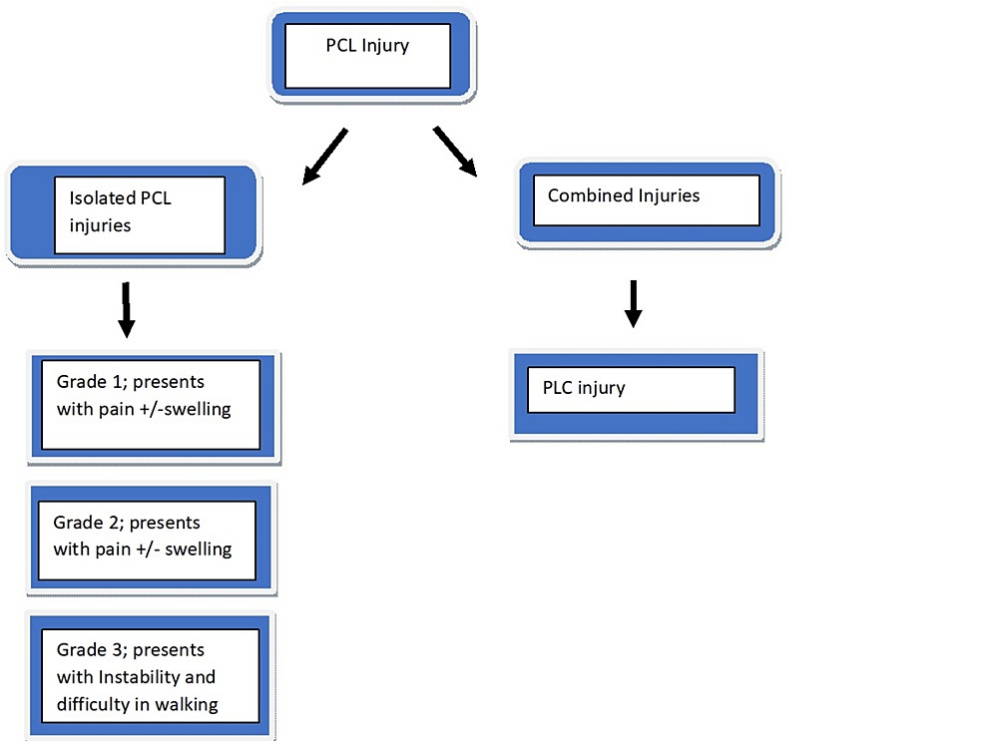
Grades Of PCL injury + = Presents with; - = Presents without; PCL: Posterior cruciate ligament; PLC: Posterolateral corner

An MRI is done in the anatomical position with 15-degree flexion and without any cast, slab, or translational force to determine the grade of PCL tears. A grade 1 partial tear with posterior translation ranges from 1 mm to 5 mm. The femoral condyles are still in front of the tibia. Grade 2 posterior tibial translation is 6 mm to 10 mm (total isolation), a complete PCL tear without any additional harm. The femoral and anterior tibial condyles are flush. In grade 3 (complete PCL with concurrent capsular and/or ligamentous injury), the posterior tibial translation is >10mm. The tibia is behind the femoral condyles and may indicate a capsuloligamentous injury in the area (Figure 1) [9].

Previous research has shown that after prolonged conservative therapy, patients with isolated PCL injuries have sustainable functional scores and satisfactory subject outcomes [10]. Therefore, nonoperative care is generally accepted as the best course of treatment for asymptomatic PCL injuries. However, new research has demonstrated that conservative therapy increases the probability of osteoarthritis, which may be brought on by high-grade PCL laxity [11-13].

The key factor to take into account when addressing PCL trauma is that even if the damage is isolated or mixed, chronic or acute, classically, irrespective of the seriousness of the injury, nonoperative care is the primary choice of management for isolated PCL injuries. This was the basis for the inconsistent outcomes of PCL repair in restoring knee function, with postoperative reports of residual looseness being standard. Despite the absence of long-term results, PCL repair is becoming a more dependable treatment thanks to improved arthroscopy techniques. The PCL has innate healing properties. This is typically carried out while relaxed. For providing acute care, elevation, ice, rest, and compression are required. Initial knee bracing has successfully treated isolated PCL injuries while reducing discomfort and posterior tibial translation [14,15].

Surgical management with PCL reconstruction is a successful option for individuals with substantial PCL laxity and uncomfortable symptoms. Numerous studies have examined the clinical and functional consequences of PCL reconstruction, comparing various surgical approaches that are single compared to double-bundle or transtibial (TT) versus tibial inlay (TI), graft sources (allograft versus autograft), or isolated and multiple ligament injuries [16,17].

This review was conducted to examine several studies on PCL, their clinical outcomes, treatment, and the reasons why PCL injuries should be treated rather than left untreated. It aims to help surgeons with decision-making regarding the management of their cases.

## Review

Causes and mechanisms of PCL injury

Car accidents (45%) and sports-related injuries were the two most frequent causes of PCL injuries (40%). These injuries commonly occur in sports and car accidents, two well-known scenarios [18]. More specifically, motorcycle accidents (28%) and soccer-related injuries (12%) are the two most frequent causes of injury (25%) [19]. However, Bernhardson et al. claimed that trauma from a sporting incident was to blame for the PCL injury in 78% of patients. Dashboard injuries (35%) and falls on a flexed knee with the foot in plantar flexion were the most frequent injury mechanisms. Skiing, rugby, football, and soccer are some of the activities where PCL rips occur most frequently. Less commonly used non-contact techniques include hyperflexion and hyperextension [20].

Indications and treatment of PCL injury

In the past, isolated PCL injuries were treated non-operatively, and multi-ligamentous knee injuries or those with recurrent instability called for surgical repair. Alterations in knee biomechanics can result from chronic PCL deficits, and studies have found that 10% more patients had arthrosis. Mixed results have been seen following surgical therapy for PCL insufficiency, perhaps due to the low volume of surgeries performed, complex anatomy, and the persistent posterior gravitational pull on the PCL at rest. The degree of the damage, concomitant injuries, the patient's level of activity, and other variables all affect how the PCL injury is treated [21,22].

Non-operative Treatment

Non-surgical management is the first line of treatment for a single grade 1 or 2 PCL tear. Surgery should be considered for patients with concurrent ligamentous injuries or those who have a chronic PCL tear with persistent laxity and recurrent instability. Patients with minimal symptoms or those who have low activity demands can receive non-operative treatment for isolated grade 3 PCL injuries. In patients with low-grade PCL injuries, those with minimal activity demands, patients who can effectively compensate for posterior tibial translation through secondary stabilizers, and patients with enhanced tibial slope, non-operative treatment has been demonstrated to yield favorable outcomes. Gaining range of motion and strengthening, with a particular emphasis on quadriceps activation, are the main goals of non-operative care [23,24]. In a study by Parolie et al., who followed 25 patients with grades 1 to 3 PCL injuries for an average of 6.2 years after injury, it was discovered that there was no significant correlation between KT-1000 testing and knee satisfaction. Instead, 80% of patients reported being satisfied with their knees, and 76% rated them as being 75% to 100% better than their uninjured knees. These patients' range of motion was identical to that of the contralateral knee, and there was no loss of quadriceps diameter greater than 1 cm [25].

Operative Management

Grade 3 injuries with less than 8 mm of posterior tibial translation, displaced PCL avulsions, multiple ligament knee injuries, or chronic PCL injuries with symptoms of instability that often arise with deceleration or descending an incline are the only ones that require surgical management. Transtibial and TI are two surgical procedures that can be further classified into single-bundle and double-bundle reconstructions. Panchal et al. completed a review comparing outcomes following both approaches and found no differences between TT or TI reconstruction. Both techniques have been validated for PCL reconstruction [26-28]. Clinical research contrasting the two methods has revealed variations in postoperatively instrumented side-to-side posterior tibial translation. Although Qi et al. and Lee et al. have published high-level research demonstrating no difference in clinical outcomes and function between approaches, it is uncertain if this variation in posterior tibial translation is clinically relevant [29,30].

For a final follow-up of 3.3 years and 9.1 years, respectively, Jenner et al. and Hermans et al. studied patients who underwent PCL repair of chronic injuries using either allograft or autograft. Both studies used bone-patellar tendon-bone autograft and Achilles allograft as graft alternatives. At the ultimate follow-up of 9.1 years, Hermans et al. discovered that the Lysholm score, International Knee Documentation Committee (IKDC), and visual analog scale (VAS) functional scores had all significantly improved. At 3.3 years, 78% of patients assessed the postoperative knee as normal or almost normal, according to Jenner et al. The Tegner activity score (TAS) improved postoperatively in both investigations, and Hermans et al. discovered a significant inverse relationship between clinical results and the tibial shaft (TS), indicating that patients with an abnormal clinical exam had trouble getting back to their typical activities [31,32]. Chronic grade C PCL injuries treated surgically with an Achilles allograft were followed up on in the short term by Goudie et al. and in the medium term by Sekiya et al. At the time of the last check-up, 80% and 57% of patients, respectively, said their knees were normal or nearly normal, and 74% and 50% of patients, respectively, said they could engage in moderate to vigorous activity. A small group of patients who had reconstruction about three months after the injury were included in the study by Sekiya et al. They discovered that patients who received treatment in the acute and subacute phases had considerably better knee function and activity levels [33,34].

Results

In a retrospective study including 40 patients, it was revealed that the patients receiving PCL reconstruction surgery showed significant improvements in the IKDC score, Lysholm score, and TA level, while surgery did not increase the risk of knee arthritis deterioration. No major complications were noted, with a minimum follow-up time of two years. Approximately half of the patients returned to activities at their preinjury level [35].

A sub-analysis of seven patients with PCL subjected to reconstruction revealed that when surgery was performed less than six months after the index injury, there were better functional outcomes. Augmenting the practical outcomes of patients after surgery is successful with reconstructive surgery for PCL or PLC injury [36].

In order to compare the consequences of isolated PCL and multi-ligament injuries, an investigation was performed on patients with symptomatic PCL tears who were subjected to surgical repair. After a re-examination of 19.5 months, 182 patients were subjected to operations, comprising 118 isolated PCL reconstructions and 75 multi-ligament knee reconstructions. There were 19 revision reconstructions and 174 primary surgeries. Around 96.2% of patients were men. The surgical impediment rate was 12.4%, with multi-ligament knee reconstructions having a considerably higher risk than isolated PCL reconstructions. About 35.1% of patients had their service terminated overall owing to an infirmity. There was no discernible difference between isolated PCL and multi-ligament knee surgeries in the revision rate, which was 10.9% overall. Around 35% of the 103 sufferers who had original isolated PCL reconstructions had to be medically discharged due to ongoing knee problems, while 12.6% needed revision PCL reconstruction. The delinquency rate for primary isolated PCL reconstructions, which takes into account both revision surgery and service termination due to knee-related medical issues, was 42.7% [37].

The clinical sequel of 237 solo PCL reconstructions and 344 multi-ligament reconstructions with combined PCL reconstruction was reported by the Danish Knee Ligament Reconstruction Registry in a follow-up of one-year duration. The knee injury and osteoarthritis outcome scores (KOOS ) and TAS were obtained by the authors in order to quantify patients' subjective experiences of their outcomes. Isolated PCL reconstructions and multi-ligament reconstructions had ameliorated the KOOS from before the operation to the re-examination of one year, according to the authors. However, they were careful to point out that the improvement was not to the same extent as that seen with anterior cruciate ligament (ACL) reconstruction. Comparatively, they also found that both solitary PCL reconstructions (3%) and multi-ligament PCL reconstructions had much-reduced reoperation rates (3.4%). According to their study, a substantial proportion of postoperative site morbidity was found, with up to 35% of single PCL reconstructions and 45% of multi-ligament reconstructions being categorized as subjective failures [38].

Sixty-eight patients with a single acute PCL injury were subjected to nonoperative management and were prospectively monitored using yearly subjective surveys and sporadic objective tests. Radiographic osteoarthritis grading, measurements of joint space width and range of motion, effusion, and quadriceps strength evaluations were all included in the physical examination. The IKDC and the modified Cincinnati knee rating system (CKRS) assessments were used for subjective re-examination. After nearly 17.6 years following trauma, all 68 patients completed introspective follow-up. The average quadriceps muscle strength was 97% that of the unaffected limb, and all the sufferers could move their knees normally. Twenty-six patients had radiographs that were classified as normal overall, 13 as nearly normal, four as abnormal, and one as severely abnormal. On the basis of laxity of PCL, there was no dissimilarity in the grade of osteoarthritis in any knee compartment on radiographs. A medial joint space narrowing of more than 2 mm was present in five individuals (11%) of the group. In PCL laxity categories, no dissimilarity was seen in subjective gradings, and the mean IKDC and CKRS subjective scores at an average of 17 years following trauma were 73.4, 21.7, and 81.3, and 17.4, respectively. Comparable subjective scores were received by patients who had undergone objective re-assessment after 10 years. Long-term outcomes following an isolated PCL trauma demonstrate that patients are still agile, have adequate strength and complete range of motion in their knees, and provide positive subjective evaluations. Around 11% of people had moderate to severe osteoarthritis. Results did not vary according to PCL laxity grade [39].

According to Belk et al., patients who underwent PCL reconstruction using autograft and had 132 patients in total with a mean age at the time of surgery of 31.6 years improved by 20 on the IKDC score, 22.7 on the Lysholm score, and 3.9 on the TAS. Clinical results for patients receiving primary PCL reconstruction, either with autograft or allograft, can be anticipated to improve. Although the therapeutic relevance of this is unknown, autograft patients had decreased postoperative anterior-posterior (AP) knee laxity, and both groups' subjective outcomes significantly and similarly improved [40].

Song et al.'s study cohort consisted of 66 individuals who underwent PCL repair for chronic injuries. There were two categories of patients, out of which 30 were in the TI group and 36 in the TT group. The mean re-examination time was 148 months, and the average duration from trauma to reconstruction was 12.2 months (with a range of two to 60 months). Results were assessed using the Lysholm score, TAS, the posterior drawer test, the Telos device for laxity testing, and the occurrence of osteoarthritis. In the TT group, the mean Lysholm score was 59.9, whereas in the TI category, it was 54.5. Postoperatively, these scores increased to 89.9 and 92.1, respectively. In the TT group, the mean TAS went from 2.5 to 5.9, and in the TI category, from 2.3 to 6.0. Nineteen patients (63.3% of the TI category) and 21 patients (58.3% of the TT category) were competent to recommence their pre-injury sporting activities. Six individuals in the TT category and four in the TI category displayed grade 2 laxity in the posterior drawer test. The mean side-to-side difference decreased postoperatively to 4.1 mm and 4.2 mm, respectively, from 10.1 mm in the TT category and 10.4 mm in the TI group. Between the final re-examination results and the preoperative values, there was a considerable improvement. However, there were no discernible variations in the final follow-up results between the two groups [41].

Patients with isolated PCL injuries who had undergone repair between 2001 and 2014 were analyzed using a retrospective review. Fifteen PCL repairs in total, with an average age of 27.5 years, were performed on 14 patients. The main preoperative complaint of all the patients was knee instability. During the physical examination, it was discovered that 93% (14/15) of the knees lacked a strong endpoint during posterior drawer testing. Three of the 15 knees had TI, and double-bundle PCL reconstruction, and 12 of the 15 patients had gone through TT, single-bundle PCL reconstruction. The patient cohort in this study had no transplant failures. The IKDC form, Lysholm score, TAS, and MAS mean scores at the most recent follow-up were, in order of standard deviation: 77.3 (16.5), 83.1 (17.9), 6.13 (2.6), and 7.1 (6.0), respectively. Prior to their accident, all 14 patients were athletes. Of those, 79% (11/14) resumed their sport, and the total patient satisfaction rating was 9.2/10. Eleven (79%) of them resumed their sport, with an all-inclusive patient contentment rating of 9.2 out of 10. At the average midterm follow-up, it was noted that isolated PCL reconstruction produces positive results with immense rates of function restoration requisite for sport, and overall patient satisfaction [42].

Patients who received primary PCL reconstruction were included in a cohort study. The reported outcome measure was the KOOS. A total of 252 patients had finished the KOOS at the two-year follow-up. To assess the impact of trauma activity and multiple ligament injuries on the results of the KOOS, multiple regression analysis was utilized. Patients injured while participating in sports had markedly better outcomes at a re-examination of two years following PCL reconstruction than patients injured while participating in other activities, in accordance with both the adjusted and uncorrected regression models, taking into account all KOOS subscales. For the KOOS subscales, the adjusted perusal was: pain (13.4; 95% CI, 8.0-18.9), daily activity task (12.6; 95% CI, 7.1-18.1), sport (15.3; 95% CI, 8.0-22.5), and quality of life (13.5). Patients with isolated PCL injuries had lower ratings on the sport/recreation subscale in the unadjusted analysis (-7.9; 95% CI, -15.5 to -0.3). The adjusted analysis found that the difference was not significant. Between single-ligament injuries and multiple-ligament injuries, there were no additional noticeable changes in KOOS results [43].

Patients diagnosed with an intense or chronic PCL injury were included in a descriptive study. The authors correlated them with isolated and mixed PCL injuries and analyzed pre- and postoperative results. Fifty-five patients were enrolled in the study. The mean time amidst trauma and surgery was 27.8 to 38.0 months, and the follow-up length was 28.83 to 20.62 months. The majority of the patients (56.4%) had a single tibial-double femoral tunnel reconstruction, whereas 34.5% had a single tibial-single femoral reconstruction. In about 60% of patients, allografts were used. After PCL restoration, the average Cincinnati knee rating scale (CKRC) also improved dramatically. Prior to surgery, only 29.1% of patients had a full range of motion; this improved following surgery (92.7%) [44].

Discussion

Although the PCL restoration resulted in improved knee stability and motor function, the patient-based assessments were not adequate. Improvement of the surgical approach is required since pain related to flexion restriction seems to be a poor prognostic indicator and there is a disconnect between subjective and objective assessments. The parameters that reliably predict clinical and functional results for both operative and non-operative treatment are unknown in terms of demographic, surgical, or patient-related characteristics. Initial non-operative therapy of PCL injuries with an optional delayed PCL reconstruction is frequently advised since non-operative treatment may produce good results. However, high-quality evidence is insufficiently supportive of the ideal time for PCL reconstruction (early vs. delayed surgery) as well as the best timing of post-injury/postoperative rehabilitation, which is even more apparent in combined PCL injuries. In comparison to patients who receive anterior cruciate ligament restoration (ACLR), patients who undergo PCL reconstruction experience worse functional and patient-reported results, and a higher proportion of patients report difficulty getting back to their previous activities. After PCL reconstruction, generally positive outcomes are reported; however, higher-quality and larger-scale studies are required to provide more support for an individualized treatment strategy with the ultimate objective of restoring natural knee kinematics and facilitating a return to daily, occupational, and athletic activities [45,46]. This review presents the findings of clinical outcomes in PCL injuries. Further studies with a comparison between young and older patients are needed to clarify the clinical and functional outcomes of PCL reconstruction in different age groups.

## Conclusions

Augmenting the functional sequelae of patients after surgery is successful in reconstructive surgery for PCL and PLC injuries. With notable improvements described as consequences that surpassed minimal clinically important difference(MCID) in many patients, along with the restoration of subjective instability and the ability of patients to get back to their normal activity levels, PCL reconstruction is a well-founded surgery for patients with determined instability even after failed conservative treatment. The right care for PCL damage is more crucial than ever because of the rising need for a good quality of life and the dynamic lifestyle of today's aged population with longer life expectancies. Overall, this analysis suggests that PCL repair can be a safe treatment, but it's crucial to keep in mind that the literature currently available only includes a limited number of low-quality case studies. Before extensive clinical implementation is justified, more dependable randomized research is required.
